# Patterns and determinants of medication use by paramedics in German prehospital emergency care: a six-year multicenter analysis

**DOI:** 10.1186/s12873-026-01506-x

**Published:** 2026-02-19

**Authors:** Christian Hohenstein, Steffen Wolfgang Nix

**Affiliations:** Faculty of Medicine, Phillips-University Marburg, Baldinger Str. 1, 35037 Marburg, Germany

**Keywords:** Drug administration routes, Emergency medical technicians, Telemedicine, Emergency medicine services, Pharmaceutical preparations, Geographic variation

## Abstract

**Background:**

Medication administration is a fundamental component of paramedic-provided prehospital emergency care within a physician-based EMS-System. In Germany, the Emergency Paramedic Act expanded pharmacological competencies and authorization pathways, yet real-world utilization of medications in routine EMS practice remains insufficiently described. This study aimed to quantify medication use, characterize medication categories and administration routes, and assess temporal and regional variation across three German EMS districts.

**Methods:**

We conducted a retrospective multicenter observational study of all paramedic-led EMS missions without on-scene physician involvement between 1 January 2019 and 31 December 2024. Electronic patient care records from three mixed urban–rural EMS districts were analyzed. Outcomes included the proportion of missions with medication administration, distribution of medication classes, administration routes, six-year temporal trends, and interregional differences. Statistical analyses included descriptive measures, chi-square tests, and Cochran–Armitage trend testing.

**Results:**

Among 197,432 eligible missions, 22,340 involved medication administration (11.3%). Crystalloids (4.1%) and analgesics (3.2%) were the most frequently administered medications, followed by bronchodilators (1.4%), glucose (1.2%), and antiemetics (0.5%). Advanced medications were used in 0.9% of missions. Medication administration increased significantly from 9.4% in 2019 to 12.1% in 2024 (*p* = 0.02). Intravenous administration was predominant (76.3%). Districts with telemedical physician support demonstrated higher overall medication use compared to the district without tele-EMS (12.7% vs. 9.1%; *p* < 0.001).

**Conclusions:**

Medication use in German paramedic-led prehospital care remains concentrated on a narrow spectrum of frequently used medication categories despite expanded legal competencies and a comprehensive pharmacology curriculum. A system supervised by the EMS medical director (ÄLRD) is essential to ensure patient safety and to support the maintenance of pharmacological competencies.

## Background

Prehospital emergency medical care plays a pivotal role in early intervention, stabilization, and improving patient outcomes. Among the key interventions performed by emergency medical services (EMS) providers, medication administration has remained an essential component, particularly in time-sensitive conditions such as acute coronary syndromes, severe asthma exacerbation, hypoglycaemia, and trauma. Despite advances in prehospital treatment protocols globally, the landscape of paramedic-led pharmacological interventions continues to evolve [1, 2].

In Germany, major reform came with the adoption of the Emergency Paramedic Act (Notfallsanitätergesetz) in 2014, which introduced an extended three-year tertiary-level education program for emergency paramedics (Notfallsanitäter) instead of the two-year education program for advanced emergency medical technicians. The Emergency Paramedic Act defines the legal framework for paramedic competencies. In routine practice, however, prehospital care in Germany is predominantly based on advance or general delegation by EMS medical directors, including delegation via tele-emergency physicians. Measures under § 2a NotSanG of the new act are intended as a case-specific safeguard when no delegation-based solution is available. The Pyramid Coordination Process provides a differentiated competency framework that explicitly distinguishes between autonomously performed measures and those requiring physician delegation [3]. However, despite this expanded scope, there is limited empirical evidence on how often paramedics actually administer medications in daily operations, and which drugs are used most frequently.

Several international studies have assessed prehospital medication use, but with varying system contexts. For example, paramedic-led EMS systems in North America and Australasia - where physician involvement on scene is low - commonly report higher frequencies of medication administration by paramedics, sometimes exceeding 20% of missions [4, 5]. Conversely, in European physician-supported models, medication use by paramedics tends to be less frequent and concentrated on a limited subset of frequently used medications [6, 7]. These findings suggest that structural characteristics of EMS systems, including delegation models, physician availability, and organizational oversight, may influence pharmacological utilization in addition to educational content. Emerging telemedicine support programs further complicate the landscape by enabling remote physician oversight and expanded paramedic role in medication decisions [8]. As such, structural, regulatory, and technological factors likely contribute to real-world medication use patterns.

Understanding medication use in a German context is particularly relevant for several reasons. First, medication interventions represent a high turnover and potential risk point in the prehospital environment, where errors in dosage, route, or indication may impact patient safety [9, 10]. Second, the investment in paramedic education is substantial - ensuring that competencies are not only taught but also used is critical for workforce effectiveness. Third, regional variation in authorization was observed. However, the present study did not systematically examine potential underlying determinants such as authorization models, local governance structures, telemedicine adoption, or SOP implementation. Beyond initial education, continuing education, competency maintenance, and supervision by EMS medical directors play a decisive role in translating pharmacological competencies into routine practice and should be considered when interpreting regional variation.

Despite these important considerations, no comprehensive multicenter longitudinal study has yet examined medication administration in paramedic-led missions in Germany. Such data are crucial for assessing whether the expanded curriculum has translated into practice and for identifying system-level levers to optimise medication use.

Thus, the objectives of this study were to: (1) quantify the overall frequency of medication administration in paramedic-led missions (2), describe the distribution of major medication categories and administration routes (3), assess temporal trends between 2019 and 2024, and (4) analyse interregional variation across three representative EMS districts. Through these analyses, we aim to provide evidence to inform policy, educational programming, and EMS system design to ensure that medication competencies taught in training align with what is used in the field.

## Materials and methods

### Study design

We conducted a retrospective multicenter observational study analysing electronic prehospital care records from three German emergency medical services (EMS) districts over a six-year period (January 1, 2019, to December 31, 2024). The study followed the STROBE guidelines for observational research. The investigation focused exclusively on missions in which emergency paramedics served as the primary care providers without an emergency physician on scene.

### Setting

Germany operates a physician-based EMS model in which paramedics and emergency physicians respond depending on dispatch criteria and local protocols. Paramedics may perform medication administration autonomously when immediate physician involvement is not possible and when the intervention is necessary to prevent imminent harm, according to the Emergency Paramedic Act. The participating EMS districts represent mixed urban–rural regions with approximately 1.1 million inhabitants collectively. Two districts operated a structured tele-EMS system during the study period, while one did not, allowing comparative analysis of structural differences.

### Participants and inclusion criteria

All prehospital missions documented in the three participating EMS districts from 2019 to 2024 were screened.

Missions were included if:

- The primary care provider was a certified emergency paramedic.
- No emergency physician was present on scene at time of initial patient contact.
- A completed electronic mission record was available.

Missions were excluded if:

- A physician arrived before any intervention (“physician-first” missions),
- Documentation was incomplete, corrupted, or missing essential variables (e.g., intervention type, medication field),
- The mission represented interfacility transport or non-clinical operations.

After applying inclusion and exclusion criteria, 197,432 missions remained for analysis.

### Data sources and variables

Data were obtained from the standardized electronic documentation system used uniformly across all participating EMS districts. The system records demographics, mission category, vital signs, interventions, medications administered (name, dose, route), and timestamps. Only fully anonymized data were used.

### Medication categories

To allow consistent analysis, medications were grouped into predefined categories:

Crystalloids (e.g., balanced electrolyte solutions).

Analgesics (non-opioid and opioid analgesics).

Bronchodilators (e.g., salbutamol, ipratropium bromide).

Glucose (e.g., intravenous glucose solutions).

Antiemetics (e.g., metoclopramide, ondansetron).

Sedatives / vasopressors / other advanced medications.

Advanced medications (sedatives, vasopressors, anticonvulsants), grouped for analytical purposes without implying standardized risk classification.

Each recorded medication administration was counted as an intervention. Route of administration (intravenous, inhaled, oral/buccal, intramuscular, intraosseous) was also extracted.

### Outcome measures

The primary outcome was the proportion of missions involving at least one medication administration (“any medication use”).

### Secondary outcomes included

Frequency of specific medication categories.

Distribution of administration routes.

Temporal trends (2019–2024) in medication administration.

Interregional variation across the three EMS districts.

### Statistical analysis

Descriptive statistics were generated for all variables.

Categorical variables are presented as frequencies and percentages.

Temporal changes over the six-year period were analysed using the Cochran-Armitage trend test.

Inter-district comparisons were performed using chi-square tests. A two-tailed p-value < 0.05 was considered statistically significant. Statistical analyses were conducted using R (version 4.3.2) and Python (version 3.11).

### Data protection and governance

Due to German data protection regulations (GDPR) and contractual obligations with EMS agencies, the dataset cannot be shared publicly. Fully anonymized data may be made available upon request and subject to approval by the respective EMS authorities. All data processing and storage complied with GDPR requirements and institutional policies.

## Results

### Study population

A total of 197,432 paramedic-led missions from three EMS districts between 1 January 2019 and 31 December 2024 met all inclusion criteria and were included in the analysis. Across the study period, annual mission volumes increased slightly from 30,221 missions in 2019 to 34,587 missions in 2024. Demographic variables such as age, sex, and primary dispatch category were comparable between regions and remained stable over time.

### Overall frequency of medication administration

Across all missions, 22,340 medication administrations were documented, corresponding to an overall rate of 11.3%. The frequency of missions involving at least one medication administration ranged from 9.1% to 12.7% across the three EMS districts. Table 1 provides an overview of medication frequencies by category.

**Table 1:** Frequency of medication administration by category (2019–2024)

Medication Category	Number of administrations (*n*)	% of all missions
Crystalloids	8,061	4.1%
Analgesics	6,331	3.2%
Bronchodilators	2,774	1.4%
Glucose	2,373	1.2%
Antiemetics	1,001	0.5%
Advanced medications (sedatives, vasopressors, anticonvulsants)	1,800	0.9%
**Total medication administrations**	**22**,**340**	**11.3%**
**Total missions**	**197**,**432**	–

Note: Percentages refer to all paramedic-led missions without on-scene physician presence

Crystalloids were the most frequently administered medication (*n* = 8,061; 4.1% of all missions), followed by analgesics (*n* = 6,331; 3.2%), bronchodilators (*n* = 2,774; 1.4%), glucose (*n* = 2,373; 1.2%), and antiemetics (*n* = 1,001; 0.5%). Advanced pharmacological agents such as sedatives, vasopressors, or anticonvulsants collectively accounted for fewer than 1% of all missions (*n* = 1,800; 0.9%).

### Temporal trends (2019–2024)

The proportion of missions involving at least one medication administration increased from 9.4% in 2019 to 12.1% in 2024. A Cochran–Armitage test for trend showed a statistically significant upward trend in overall medication use across the six-year period (*p* = 0.02).

**Fig. 1:**
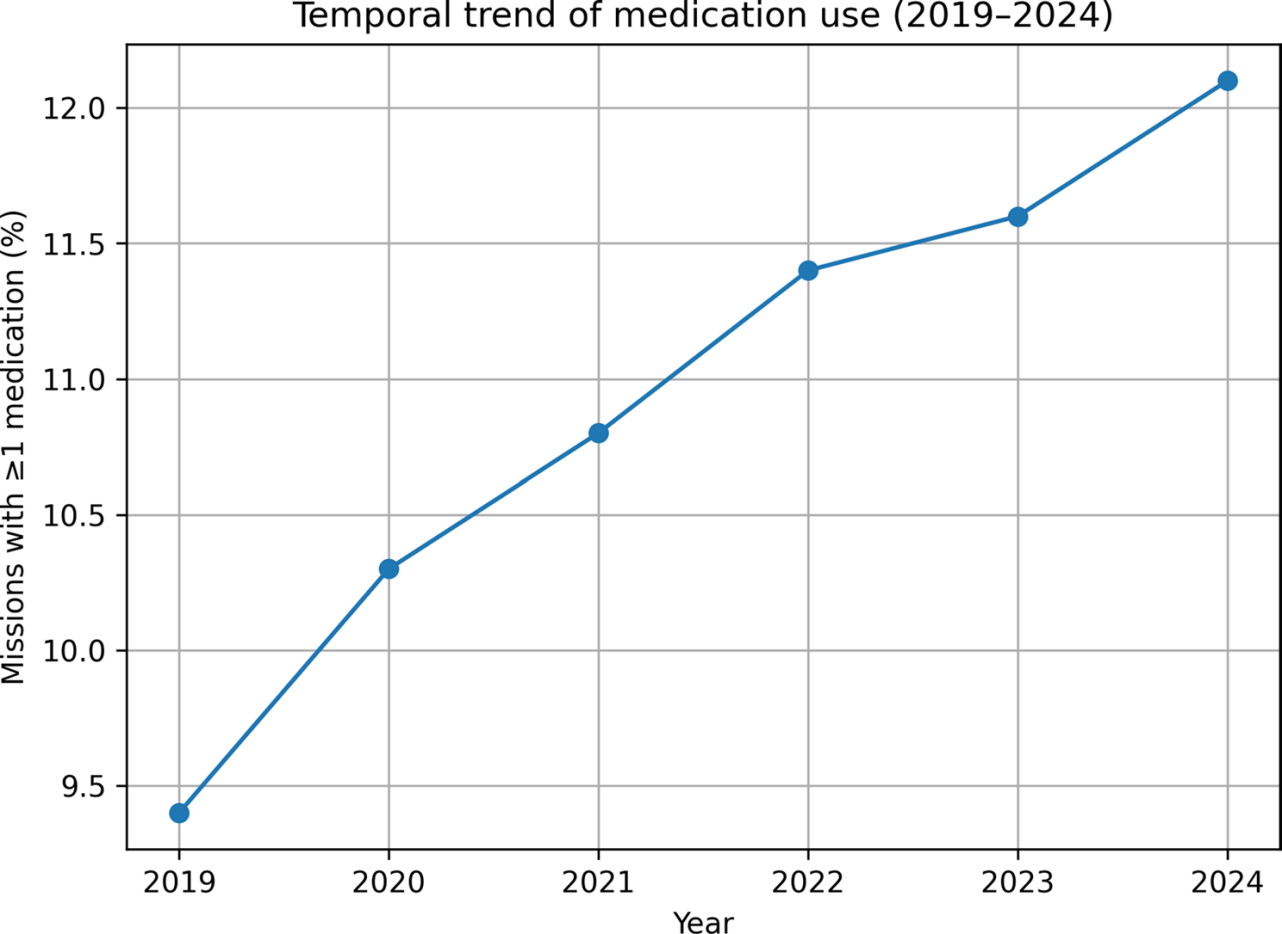
Temporal trend of medication use

The absolute distribution of medication categories changed only modestly over time, and the rank order of the most frequently used categories remained stable. Annual frequencies are summarised in Table 2, and the temporal trend is illustrated in Fig. 1.

**Table 2:** Annual frequency of medication administration (2019–2024)

Year	Total missions	Missions with ≥ 1 medication	% medication use
2019	30,221	2,845	9.4%
2020	31,004	3,204	10.3%
2021	32,118	3,485	10.8%
2022	33,587	3,841	11.4%
2023	35,915	4,176	11.6%
2024	34,587	4,789	12.1%
**Total**	**197**,**432**	**22**,**340**	**11.3%**

Note: Cochran–Armitage test for linear trend in proportion of missions with ≥ 1 medication administration across years: *p* = 0.02

All major medication categories demonstrated modest increases over time, with the most pronounced rise observed in analgesic and bronchodilator administration.

The relative distribution of medication categories remained stable, and no year showed major deviations in drug class proportions. Table 2 summarises the annual medication frequencies.

### Administration routes

**Fig. 2:**
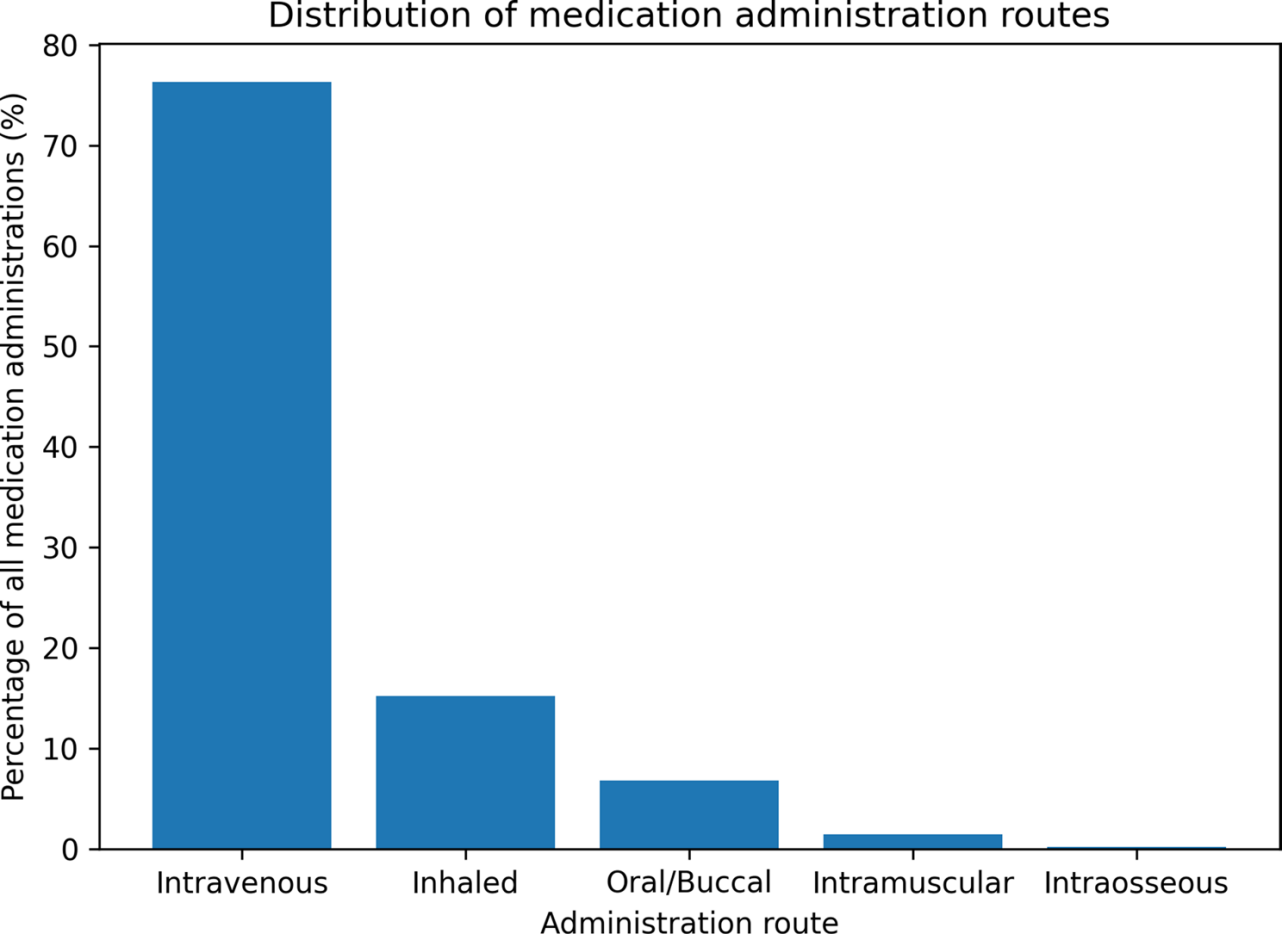
Admininstration routes

Of the 22,340 medication administrations, 76.3% were given intravenously, 15.2% via inhalation, 6.8% orally or buccally, 1.5% intramuscularly, and 0.2% intraosseously. The distribution of administration routes did not show a relevant temporal shift; no formal trend testing was performed for route of administration. The overall distribution is shown in Table 3; Fig. 2.

**Table 3:** Distribution of medication administration routes

Administration Route	Number of administrations (*n*)	Percentage of all medication administrations
Intravenous (IV)	17,057	76.3%
Inhaled	3,396	15.2%
Oral / Buccal	1,519	6.8%
Intramuscular (IM)	335	1.5%
Intraosseous (IO)	33	0.2%
**Total**	**22**,**340**	**100%**

Note: No formal statistical trend testing was performed for route of administration

Route-of-administration patterns remained consistent across the study period, with no clinically significant temporal shifts. Figure 2 illustrates the distribution of administration routes across all missions.

### Medication categories

#### Crystalloids

Crystalloids were administered in 8,061 missions (4.1%) and represented the largest medication category. Annual use increased from 3.6% in 2019 to 4.4% in 2024.

#### Analgetics

Analgesics were administered in 6,331 missions (3.2%). Non-opioid analgesics (e.g., metamizole) were more common than opioid analgesics (e.g., fentanyl, morphine). Annual frequencies rose from 2.7% to 3.5% across the study period.

#### Bronchodilators

Bronchodilators were used in 2,774 missions (1.4%). Salbutamol was the most frequently administered agent, followed by ipratropium bromide. Annual usage increased steadily in line with respiratory-related dispatches.

#### Glucose

A total of 2,373 administrations (1.2%) were recorded for antihypoglycemic therapy. Intravenous glucose accounted for the majority of cases, with oral glucose solutions used less frequently.

#### Antiemetics

Antiemetic medications were administered in 1,001 missions (0.5%). Metoclopramide was the most frequently used agent, followed by ondansetron.

#### Advanced medications (sedatives, vasopressors, anticonvulsants)

Advanced medications were rare with 1,800 administrations (0.9%). This category included ketamine, midazolam, adrenaline (in subanaphylactic indications), and other medications requiring careful monitoring. Advanced drug use remained below 1.2% in all years.

### Interregional variation

Medication administration varied across EMS districts:

- District A (with tele-EMS support): 12.7%.
- District B (with tele-EMS support): 10.8%.
- District C (without tele-EMS support): 9.1%.

Table 4 provides an overview over the interregional variation.

**Table 4:** Interregional variation in medication use

EMS District	Tele-EMS Support	Total missions	Missions with ≥ 1 medication	% medication use
District A	Yes	62,110	7,891	12.7%
District B	Yes	68,741	7,428	10.8%
District C	No	66,581	6,021	9.1%
**Total**	–	**197**,**432**	**22**,**340**	**11.3%**

Note: Chi-square test comparing proportion of missions with ≥ 1 medication administration between districts: *p* < 0.001

Differences were statistically significant (*p* < 0.001).

Districts with telemedical support showed consistently higher medication frequencies across all drug categories. Crystalloid and analgesic use differed by up to 1.3% points between districts, while bronchodilator and glucose administration showed smaller relative differences. Advanced medication use also varied, but remained below 1.1% in all regions.

### Mission types associated with medication use

Medication administration occurred more frequently in:

- respiratory distress missions,
- trauma-related calls,
- cardiovascular emergencies,
- neurologic complaints (e.g., seizures, stroke mimics), and.
- hypoglycemia missions.

Administrative mission categories (e.g., “general weakness,” “nonspecific malaise”) exhibited lower medication usage rates.

In summary, medication administration occurred in just over one in ten paramedic-led missions, with a significant increase over time (*p* = 0.02) and significant variation between districts (*p* < 0.001). Crystalloids and analgesics were consistently the most frequently used medication categories, and intravenous administration was the dominant route.

### Missing data

Missing or incomplete documentation accounted for < 0.4% of all missions. These cases were excluded from analysis according to predefined criteria. No variables essential to the primary outcome exceeded 1% missingness.

## Discussion

This six-year multicenter observational study provides the most comprehensive assessment to date of real-world medication use by paramedics in German prehospital emergency care. Previous studies from individual German regions have reported substantially lower medication rates [11] and also low measures and interventions by paramedics [12]. Other studies have demonstrated frequencies comparable to those observed in the present analysis [13, 14], and others have discussed variability in the implementation of recommended skills [15]. Differences between studies may reflect stratification of clinical responsibility, although low overall incidence of such scenarios may also contribute. In this context, medication administration in more than one in ten paramedic-led missions, with a significant increase over time, indicates a focused pattern of pharmacological interventions rather than underutilization. The majority of administered medications were frequently indicated agents such as crystalloids, analgesics, bronchodilators, and glucose, consistent with typical mission profiles. Advanced pharmacological interventions accounted for a small proportion of missions, reflecting the integration of emergency physicians in high-acuity care within the German EMS model. This discussion contextualizes the findings within the existing literature, explores structural explanations for observed patterns, and outlines how system-level frameworks may support sustained competency application in prehospital emergency care.

### Principal findings

Three major insights emerged from the analysis. First, medication administration by paramedics followed a focused pattern in both frequency and medication spectrum within the investigated mission subset, consistent with a system that allocates higher-acuity pharmacological escalation to physician-supported pathways. Second, although a statistically significant increase in medication use was observed over time, this trend remained moderate, suggesting gradual and sustained integration of pharmacological interventions into routine practice. Finally, intravenous administration predominated across all years, reflecting stable procedural practice patterns; the limited use of certain advanced pharmacological agents in this dataset is best interpreted in light of mission selection and physician integration rather than as an indicator of insufficient competence.

These findings, which include medication administrations by paramedics prior to physician arrival, align with previous European studies reporting lower frequencies of advanced prehospital pharmacological interventions in physician-supported EMS systems [6, 16–18].

### Medication use in the context of German EMS structure

Germany operates a hybrid EMS model in which paramedics hold expanded competencies while emergency physicians remain closely integrated into frontline response [19–21]. Within physician-based systems, pharmacological decision-making is typically embedded in structured delegation pathways and physician availability, which shapes the distribution of responsibilities between professional groups. In this organizational context, the medication patterns observed in the present study are compatible with a functional division of labor and standardized clinical governance rather than differences in professional capability.

This distinction is well documented in international comparisons. Physician-led EMS systems, such as those in Germany, Austria, and France, consistently report lower rates of paramedic-administered medications [6, 17, 22–25], whereas Anglo-American EMS systems demonstrate higher utilization of both basic and advanced pharmacological interventions [23, 26–29]. Accordingly, the medication frequencies observed in this study are consistent with the structural logic of the German EMS model.

The observed distribution further mirrors international findings indicating that analgesia, bronchodilation, and glucose management represent the most common pharmacological interventions performed by paramedics [30–37].

### Rarity of advanced pharmacological interventions

Advanced pharmacological agents, including sedatives, vasopressors, and anticonvulsants, accounted for fewer than 1% of missions in this dataset. This finding is consistent with earlier studies reporting limited use of high-complexity drug classes among paramedics in physician-based EMS systems [38–40].

From a system perspective, this may reflect stratification of clinical responsibility within the physician-based EMS system. However, it is also possible that clinical scenarios requiring such interventions are rare overall. The present data do not allow differentiation between these explanations. Within this framework, the observed patterns emphasize the importance of system-level mechanisms—such as structured delegation, continuing education, simulation training, and medical director oversight—to support competency maintenance for low-frequency, high-acuity interventions. While considerations regarding skill retention are relevant, they cannot be directly inferred from the present data and warrant further investigation.

### Temporal increase in medication use

The significant rise in medication administration between 2019 and 2024 may reflect willingness to initiate pharmacological interventions. Studies demonstrate improved decision-making and higher compliance with medication guidelines, especially when telemedical oversight is available [8, 41].

In addition, evolving SOP frameworks and competency certification processes may have supported more consistent application of pharmacological competencies over time; however, these factors were not systematically examined in the present study.

### Education–practice gap

A central observation of this study is the apparent difference between the breadth of pharmacological training provided during paramedic education and the range of medications administered in the missions analyzed; however, this finding must be interpreted cautiously in light of the study’s inclusion criteria. This pattern is consistent with system-integrated practice in a physician-based EMS model and reflects selective application within defined clinical contexts rather than a general deficit in training or capability [21]. The phenomenon highlights the relevance of several areas. From a skill-retention perspective, competencies that are taught but rarely applied in clinical practice are susceptible to decay over time. While this relationship is well established for procedural skills, such as airway management, analogous mechanisms are likely relevant for pharmacological decision-making [42–45]. Equivalent data for pharmacology exposure are limited but analogous principles likely apply. Simulation-based training may partially mitigate this gap by maintaining cognitive familiarity and procedural confidence. Medication errors remain a recognized risk in EMS [10, 46–48]. However, simulation cannot fully reproduce the time pressure, uncertainty, and contextual complexity of real prehospital missions. From a patient-safety standpoint, limited exposure to advanced pharmacological interventions may underscores the importance of errors in rare but high-stakes scenarios, underscoring the importance of structured reinforcement strategies.

The study design deliberately focused on paramedic-led missions without on-scene physician involvement in order to characterize pharmacological practice within this specific operational context. In the German EMS system, high-acuity emergencies—such as severe shock, refractory seizures, or advanced airway compromise—are commonly managed through early physician dispatch or rapid physician arrival and were therefore not the primary focus of the present analysis. Within this defined mission subset, advanced pharmacological agents were used infrequently, reflecting appropriate task allocation and system-based stratification of clinical responsibility rather than limited paramedic capability. Medication use in scenarios with delayed or unavailable physician arrival represents an important complementary aspect of prehospital pharmacological practice and warrants further investigation in future studies.

### Comparison with international data

International EMS systems report medication use frequencies substantially higher than those observed here. Canadian paramedic services: 20–25% of missions include medication administration [4, 49] and Australian paramedic-led EMS: 30–40% [50] whereas U.S. EMS data show 30%+ medication administration in national datasets [51].

These comparisons must be interpreted in light of system design:

Anglo-American systems emphasize standing orders and paramedic autonomy in contrast to German and Central European systems that involve routine physician availability, reducing the number of paramedic-led pharmacological decisions.

Thus, lower frequencies in Germany should be interpreted in the context of system design and physician integration rather than as indicators of underperformance.

### Considerations future design and system research

The findings of this study provide descriptive insights into how existing system-level structures shape pharmacological practice in paramedic-led prehospital care. Rather than indicating unmet needs or deficits, the observed patterns illustrate how delegation frameworks, telemedical physician support, standardized SOPs, and medical director oversight collectively enable focused and context-appropriate medication use within a physician-based EMS model.

In this context, it highlights regional differences that may be related to organizational implementation. The higher medication use observed in districts with telemedical physician support suggests that real-time clinical oversight may facilitate decision-making and support consistent application of pharmacological competencies within established authorization pathways.

The selective use of advanced pharmacological agents further underscores the relevance of system design in allocating clinical responsibility. Existing structures appear to appropriately channel high-acuity pharmacological escalation toward physician-supported pathways, while allowing paramedics to apply frequently indicated medications autonomously in routine missions.

From an organizational perspective, these findings emphasize the role of continuing education, simulation training, and structured feedback mechanisms in supporting competency maintenance for low-frequency, high-acuity interventions. Rather than prescribing specific models, the present results suggest that system-level integration and governance are key factors in sustaining safe and effective medication practice.

Future research may build on these observations by examining how different organizational configurations—including telemedical support models, delegation practices, and continuing education frameworks—interact to influence medication use, decision-making, and patient-centered outcomes across the full spectrum of EMS missions.

### Strengths and limitations

This study has several strengths, including the largest multicenter dataset on paramedic-led medication use in Germany to date, a six-year longitudinal design, and uniform electronic documentation across all participating EMS districts. These features enhance internal consistency and allow robust temporal and regional comparisons.

Several limitations must be acknowledged. First, the retrospective observational design precludes causal inference, and no linkage to clinical outcomes was possible. Second, the analysis was restricted to paramedic-led missions without on-scene physician presence. As a result, high-acuity cases—where advanced pharmacological interventions may be more common—were systematically excluded, potentially leading to an underestimation of overall medication use and paramedic autonomy. Finally, although missing data rates were low, documentation inaccuracies and underreporting cannot be fully excluded, particularly in high-stress scenarios.

Differences in population density, hospital availability, and EMS organizational models between regions were not formally analyzed and may have influenced medication administration patterns.

Although regional variation was observed, SOP content, delegation practices, and continuing education structures were not systematically compared and therefore cannot be conclusively linked to the observed differences. However, all three EMS districts operate under the supervision of the same EMS medical director, which implies a high degree of procedural harmonization. Core SOPs and authorization frameworks are largely identical across regions, suggesting that observed variation is unlikely to be explained by fundamental differences in formal protocols alone.

The category “advanced medications” was used for analytical purposes only. We acknowledge that this classification is not standardized and may appear arbitrary. Clinical complexity depends primarily on patient condition, indication, and monitoring requirements rather than medication class alone. Therefore, this term should not be interpreted as a risk-based or hierarchical categorization.

The inclusion of crystalloid infusions may inflate overall medication frequencies. However, crystalloids represent a relevant therapeutic intervention in routine prehospital care and were therefore deliberately included to reflect real-world practice.

### Differentiation between authorization pathways

A major limitation of this study is that we were unable to differentiate between pre-delegated pharmacological interventions and measures performed autonomously under § 2a NotSanG. The documentation system does not systematically capture the legal or organizational authorization pathway underlying each medication administration.

Consequently, it is not possible to determine to what extent higher medication frequencies in districts with telemedical support reflect pre-delegated measures, real-time physician supervision, or autonomous emergency actions. This limits interpretation regarding paramedic autonomy and the specific impact of telemedical support on pharmacological decision-making.

### Selection, documentation and confounding biases

A selection bias arises from focusing on paramedic-led missions without on-scene physician presence: High-acuity cases, which typically require physician involvement, are systematically excluded, potentially artificially lowering the observed medication administration rates (11.3%) and underestimating paramedics’ true autonomy in practice. This reflects the hybrid structure of German EMS but risks rendering the sample unrepresentative of all mission types.

A documentation bias, common in retrospective analyses of electronic patient records (EPRs). Although the missing-data rate was low (< 0.4%), underreporting of medication administrations (e.g., routine oral doses) or inconsistencies in categorization (e.g., “other advanced medications”) could distort frequencies. This is particularly relevant in EMS contexts, where documentation occurs under time pressure and error rates for medication reports can reach 10–15%. Sensitivity analyses to validate documentation accuracy are absent, which future studies should address.

A confounding bias exists due to unadjusted covariates such as mission severity (e.g., NACA score), patient demographics, or seasonal fluctuations, which could modulate medication use.

### Future research

Future studies should:

- Link medication use to patient outcomes.
- Examine cognitive and organisational factors influencing medication decisions.
- Evaluate the impact of telemedical support on advanced pharmacological interventions.
- Assess optimal frequency thresholds for pharmacology skill retention in EMS.
- Analyse medication accuracy and error rates in relation to training and system structure.
- Prospective cohort studies with validated documentation tools and multivariate adjustment.

## Conclusion

This multicenter observational study provides a comprehensive overview of medication use in German paramedic-led prehospital emergency care over a six-year period. Medication administration occurred in approximately one in ten missions and remained concentrated on a narrow spectrum of frequently used medication categories.

Advanced pharmacological interventions were rare, and relevant regional variation in medication use was observed. The present study did not examine authorization models or delegation pathways, precluding causal interpretation. Gradual increase in medication administration occurred over time.

These findings describe current real-world practice in paramedic-led missions without on-scene physician involvement. Further studies examining system characteristics, authorization models, and telemedical support structures are needed to better understand determinants of regional variation and advanced medication use.

## Data Availability

Due to legal and ethical restrictions related to German data protection law (GDPR) and regional EMS regulations, the underlying operational EMS datasets cannot be made publicly available. Data, which will be fully anonymized, can be requested by mail from the authors.
